# Self-Objectification and Personal Values. An Exploratory Study

**DOI:** 10.3389/fpsyg.2017.01055

**Published:** 2017-06-23

**Authors:** Chiara Rollero, Norma De Piccoli

**Affiliations:** ^1^Facoltà di Psicologia, Università degli Studi eCampusNovedrate, Italy; ^2^Dipartimento di Psicologia, Università degli Studi di TorinoTurin, Italy

**Keywords:** self-objectification, body shame, body surveillance, personal values, gender

## Abstract

Self-objectification occurs when individuals treat themselves as objects to be viewed and evaluated based upon appearance. Literature has largely elucidated links between self-objectification and damaging outcomes in both men and women. The purpose of the present study was to extend past research on the antecedents of self-objectification. We were interested in the role played by specific ideological components, i.e., higher order personal values (self-enhancement, conservation, self-transcendence, and openness to change), in influencing the degree to which individuals internalize the objectifying perspective of the Western cultural milieu, i.e., self-objectify. Undergraduate participants (*N* = 371, 76.8% women) completed measures of self-objectification (i.e., body surveillance and body shame), and endorsement of higher order values. Regression analyses demonstrated that self-enhancement is linked to higher self-objectification in both men and women, whereas conservation is related only to women’s body surveillance. Self-transcendence seemed to act as a buffer against men’s body surveillance, whereas openness to change resulted as a buffer against women’s body surveillance. Implications are discusses.

## Self-Objectification: Consequences and Antecedents

According to the social constructionist account of feminist analyses, in Western societies the female body is socially constructed as an object to be regarded and evaluated. The objectification theory (Fredrickson and Roberts, 1997) posits that women often are looked at as objects by society, with a sexual focus being placed on their bodies rather than on their abilities. The ubiquity of these objectification experiences socializes women to internalize an observer perspective upon their body. This process is called self-objectification and occurs when women think about and treat themselves as objects to be regarded and evaluated based upon appearance (Fredrickson and Roberts, 1997; McKinley, 2011).

Since the foundational work of Fredrickson and Roberts (1997), literature has largely demonstrated the damaging psychological corollary of self-objectification. Experimental research has shown that heightened self-objectification promotes general shame, appearance anxiety, drive for thinness, hinders task performances and increases negative mood (Moradi and Huang, 2008; Gervais et al., 2011; Rollero, 2013; Tiggemann, 2013). Consistently, correlational studies have found that self-objectification is related to appearance anxiety, body shame, positive attitudes toward cosmetic surgery, depression, sexual dysfunction and various forms of disordered eating (e.g., Miner-Rubino et al., 2002; Calogero, 2009; Calogero et al., 2010; Peat and Muehlenkamp, 2011; Tiggemann and Williams, 2012). Most correlational studies have been cross sectional, but some longitudinal data are available as well and report similar outcomes (McKinley, 2006).

Even if objectification theory was developed in reference to women’s experiences, research has explored the applicability of this framework to investigate men’s experience as well. Studies have shown that men report lower self-objectification than do women, but young male adults are becoming progressively more worried about their physical aspect (Weltzin et al., 2005; Moradi and Huang, 2008). This seems to be related to the growing tendency to objectify men’s bodies in Western societies, which increases body image concerns among men (Johnson et al., 2007; Daniel et al., 2014). In line with findings about women, men’s self-objectification is correlated with lower self-esteem, negative mood, worse perceived health and disordered eating (Calogero, 2009; Rollero, 2013; Register et al., 2015; Rollero and De Piccoli, 2015). Moreover, self-objectification processes have been taken into account to explain drive for muscularity, excessive exercise and steroid use in men (Daniel and Bridges, 2010; Parent and Moradi, 2011). In sum, a great number of studies grounded in objectification theory have elucidated links between self-objectification processes and relevant psychological outcomes both in female and in male populations.

Fewer studies have driven the attention to the potential antecedents of self-objectification. Most of them emphasize the role played by mass media: literature has clearly demonstrated the relationship between viewing objectified media models and both men and women’s self-objectification (e.g., Groesz et al., 2002; Tiggemann, 2003; Grabe et al., 2008; López-Guimerà et al., 2010; Rollero, 2013; Vandenbosch and Eggermont, 2014). The internalization of the objectifying messages from the media leads individuals to self-objectify and guides the perception of their worth (Thompson and Stice, 2001; Vandenbosch and Eggermont, 2012; Karazsia et al., 2013).

Recently, some authors have pointed out the necessity to address the ideological antecedents of self-objectification. In their experimental studies, Calogero and Jost (2011) found that women exposed to specific ideology, i.e., sexist attitudes, increase their level of self-objectification. They conclude that self-objectification can be considered as a consequence of an ideological pattern that justifies and preserves the societal status quo.

Teng et al. (2016a), with a sample of Chinese women, showed that women’s values play a role in fostering a self-objectifying perspective, besides other sociocultural and interpersonal predictors. By means of an experimental study, these authors induced materialism and found that “certain situational cues that do not contain any explicit information about the physical body could give rise to self-objectification” (Teng et al., 2016a, p. 226). Thus, they demonstrated that materialism can trigger self-objectification tendencies. In line with this research, Teng et al. (2016b) in their study with Chinese subjects showed that the more materialistic women are, the more likely that they adopt on an objectifying gaze upon themselves and show more monitoring of their body.

Despite these two recent studies and few exceptions (Loughnan et al., 2015 for the impact of culture on male and female self-objectification; Myers and Crowther, 2007 for the role of feminist beliefs and Hurt et al., 2007 for the role of feminist identity) to the best of our knowledge no other research has explored the role played by specific ideological components, such as personal values, in the development of self-objectification. However, according to Howard (1985), values play an important role in shaping people attitudes and behaviors.

The present study addresses this issue, considering that a broader pattern of personal values may influence the degree to which both male and female individuals accept and internalize the objectifying perspective of Western cultural milieu, i.e., self-objectify.

## Conceptualizing Values: The Schwartz Value Theory

Schwartz (1992, 1994) defined values as desirable, abstract, trans-situational goals that vary in importance and serve as guiding principles in the life of a person. According to Schwartz (1992), a set of basic values is recognized in all societies and is organized into a coherent system that underlies attitudes and behaviors. This coherent structure comes from the social and psychological conflict or congruity between values that individuals feel when they make decisions (Schwartz, 1992, 2006). The classic version of the Schwartz value theory (Schwartz, 1992) identified 10 basic human values grouped in 4 higher order values. Recently, Schwartz et al. (2012) proposed a refined theory, which distinguished 19 more narrowly defined values, grouped in the same 4 higher order value. Such higher order values are: self-enhancement, intended as power over people and achievement of personal success through demonstrating competence according to social standards; openness to change, defined as the extent to which people are motivated to follow their own intellectual and emotional interests in uncertain directions, through the cultivation of their own ideals and ability; self-transcendence, referred to importance given to concern for others, broadly defined, and to spiritual life, meaning in life, unity with nature, and inner harmony; conservation, which combines conformity, security, and tradition, and refers to the extent to which people are motivated to preserve the status quo and the certainty it provides (Schwartz, 1992; Cieciuch et al., 2014).

According to the Schwartz value theory (Schwartz, 1992), these four values form two basic bipolar dimensions. The first dimension is called *openness to change versus conservation* and groups values in terms to the extent to which they motivate individuals to pursue their freedom in unpredictable ways versus to maintain the status quo in relationships with other, institutions, and traditions. The second basic dimension is called *self-enhancement versus self-transcendence* and combines selfish concerns, power achievement and hedonism in opposition to universalism and benevolence values. Finally, the theory specifies that self-enhancement and openness to change are personal focused values, as they refer to individual dimensions, whereas conservation and self-transcendence are social focused values, as they deal with the relationships between the individual and his or her social context and environment (Schwartz, 1992; Cieciuch et al., 2014).

The theory of Schwartz has been tested in extensive cross-cultural research (e.g., Bardi et al., 2009; Davidov, 2010; Cieciuch et al., 2014) and in relation to different areas, such as social and political activism (e.g., Caprara et al., 2012; Talò and Mannarini, 2015; Vecchione et al., 2015), group relations (e.g., Levin et al., 2015), working life (e.g., Sortheix et al., 2015), parenting style (Knafo and Schwartz, 2003), consumers’ behaviors (e.g., Choi et al., 2015). Moreover, basic values are quite stable across time (Schwartz, 2006), changing little even in the face of many life transitions (Bardi et al., 2014).

To sum up, supported by numerous experiments and field studies, values seem predictive of content specific attitudes and behaviors, due to their stable higher order cognitive representation of human motivations and life orientations. Since they find expression in all domains of life and therefore underlie all attitudes and opinions (Schwartz et al., 2010), in the present study we suggest that values affect the attitude people have toward the importance of their appearance. Specifically, we argue that self-objectification might be a consequence of a set of values that implicitly considers body appearance as an essential element for personal success, self-worth, and social acceptance.

## Current Study

The purpose of the present study was to extend past research on the antecedents of self-objectification. We were interested in the role played by specific ideological components, i.e., higher order personal values, which may influence the degree to which individuals accept and internalize the objectifying perspective of the Western cultural milieu, i.e., self-objectify.

In line with literature on self-objectification (see Tiggemann, 2013), in the present study self-objectification was operationalized through the construct of objectified body consciousness (McKinley, 2011), which refers to the degree to which people think about and treat their body as an object. Two main components of this construct are usually measured: (a) body surveillance — viewing the body as an outside observer, and (b) body shame — feeling shame when the body does not conform to cultural standards. Finally, following the suggestion of Moradi and Huang (2008) referring to the necessity of evaluating in empirical research, rather than assuming, construct equivalence for men and women, both males and females were involved in the present study.

Based on the above described literature, we expected that:

Hypothesis 1: Self-enhancement should be linked to high self-objectification (i.e., body surveillance and body shame) in both men and women. Since it refers to the achievement of personal success through societal standards (Schwartz, 1992) and in Western cultures societal standards concerning appearance promote men and women’s self-objectification (Fredrickson and Roberts, 1997; Daniel et al., 2014), individuals who attribute priority to self-enhancement are expected to be more involved in self-objectification processes.
Hypothesis 2: Conservation should be related to self-objectification (i.e., body surveillance and body shame) only in the female population. Since it refers to the maintenance of traditions (Schwartz, 1992) and traditional sexist ideologies consider the pursuit of beauty a duty mainly for women than for men (Glick et al., 2005; Fikkan and Rothblum, 2012), female participants who are concerned with the necessity to preserve traditional values are supposed to be more engaged in self-objectification processes.
Hypothesis 3: Self-transcendence should be linked to lower levels of self-objectification (i.e., body surveillance and body shame) in both men and women. Since individuals showing high self-transcendence attribute priority to concern for others and for spiritual life (Schwartz, 1992), they should be less sensitive to self-objectification processes, which instead drive the attention to the monitoring of one’s body and appearance (Fredrickson and Roberts, 1997).
Hypothesis 4: Openness to change should be related to reduced self-objectification (i.e., body surveillance and body shame) in both men and women. Since openness to change implies the motivation to follow personal interests and ideas (Schwartz, 1992) and self-objectification represents the internalization of a cultural perspective (Fredrickson and Roberts, 1997), individuals who pursue their own emotional and cognitive freedom should be less prone to internalize the objectifying perspective.

## Method

### Participants

Participants were 371 Italian undergraduates in Psychology (76.8% female, mean age = 21.1 years, *SD* = 2.03, range 18–29). Their mean body mass index was 21.20 (*SD* = 3.37). Specifically, 90.5% of participants were at a healthy weight (BMI range 18.40–24.90), whereas the remaining 9.5% were overweight (BMI range 25–30.85). Participants were recruited in several classrooms during the break (University of Turin). No fulfillment of course requirements was given in exchange for their participation.

### Ethics Statement

This study was carried out in accordance with the Declaration of Helsinki, the Charter of Fundamental Rights of the European Union, the European Data Protection Directive (95/46/EC and following updates), and Italian laws on privacy and data protection (L. 196/2003). All participants participated voluntarily and were free to fill in or not the questionnaire.

The questionnaire used for data collection included a cover sheet explaining the research aim, the voluntary nature of participation, the anonymity of the data, and the elaboration of the findings.

This study is part of a larger research project for which has been requested, and obtained, the approval from the Bioethics Committee of the University of Turin.

### Measures

Data were gathered by a self-reported questionnaire which took about 15 min to be filled in. The following variables were assessed:

#### Self-objectification: Body Shame

The Body Shame subscale of the Objectified Body Consciousness Scale (McKinley and Hyde, 1996) was administered. It is an eight item scale used to measure self-objectification and feelings of shame when one’s body does not conform to cultural standards. Participants responded to a 7-point scale ranging from “strongly disagree” to “strongly agree” (Cronbach’s α = 0.83). (e.g., “When I can’t control my weight, I feel like something must be wrong with me”).

#### Self-objectification: Body Surveillance

The Body Surveillance subscale of the Objectified Body Consciousness Scale (McKinley and Hyde, 1996) was also used. It measures the frequency with which participants monitor their physical appearance and consists of eight items on a 7-point scale ranging from “strongly disagree” to “strongly agree” (Cronbach’s α = 0.84) (e.g., “I rarely think about how I look” – reversed item).

#### Personal Values

Based on the Schwartz’s Values Survey (Schwartz, 1992, 2006) respondents rated 56 values “as a guiding principle in my life,” using a 5-point scale ranging from “opposed to my values” to “of supreme importance.” Following the Schwartz’s model, items were grouped into four subscales, referring to the higher order values: self-enhancement (α = 0.81), conservation (α = 0.72), self-transcendence (α = 0.84), and openness to change (α = 0.79).

#### Body Mass Index

Participant reported their height and weight, which were used to calculate BMI (kg/m^2^).

## Results

All statistical analyses were carried out using the SPSS 21.0 software.

Descriptive statistics are reported in **Table 1**.

**Table 1:** Descriptive analyses and correlations of the studied variables.

	Min	Max	*M (SD)*	1	2	3	4	5
(1) Body shame	1	6.75	3.15 (1.24)					
(2) Body surveillance	1.38	7	4.52 (1.08)	0.62^∗∗^				
(3) Self-enhancement	1.70	4.90	3.06 (0.53)	0.20^∗∗^	0.24^∗∗^			
(4) Conservation	2.24	4.53	3.30 (0.44)	0.18^∗∗^	0.10	0.33^∗∗^		
(5) Self-transcendence	2.22	5	3.98 (0.45)	0.00	–0.07	0.14^∗^	0.52^∗∗^	
(6) Openness to change	2.36	5	3.90 (0.49)	–0.06	–0.10	0.36^∗∗^	0.21^∗∗^	0.54^∗∗^

*^∗^*p* < 0.05, ^∗∗^*p* < 0.01.*

Before testing the relation between personal values and self-objectification, *T*-tests were performed to assess women/men differences concerning body shame, body surveillance and values. As presented in **Table 2** women outscored men on both shame and surveillance. Concerning values, men attributed higher priority to self-enhancement and lower priority to conservation and self-transcendence than did women. No significant difference between women and men emerged in relation to openness to change.

**Table 2:** Differences between men and women on self-objectification and personal values.

	Men: *M* (*SD*)	Women: *M (SD)*	*T* value	Sig.
Body shame	2.64 (0.96)	3.30 (1.27)	–4.32	<0.001
Body surveillance	4.08 (1.13)	4.65 (1.03)	–4.04	<0.001
Self-enhancement	3.28 (0.62)	2.98 (0.48)	4.34	<0.001
Conservation	3.21 (0.44)	3.33 (0.43)	–2.15	<0.05
Self-transcendence	3.86 (0.44)	4.02 (0.44)	–2.93	<0.01
Openness to change	3.98 (0.50)	3.87 (0.49)	1.81	n.s.

The hypothesized relationships were tested by means of two hierarchical regression models. Each dimension of self-objectification, i.e., body shame and body surveillance, was regressed onto the four higher order values. We controlled for the effect of BMI, given that this variable may affect self-objectification (Tiggemann and Lynch, 2001; Rollero and De Piccoli, 2015). The analyses were carried out for male and female samples separately.

As shown in **Table 3**, values did not play any relevant role in predicting men’s body shame. The unique significant independent variable was BMI, demonstrating that men who are overweight experienced higher shame than those at a healthy weight. In case of the female population, two values fostered body shame, i.e., self-enhancement and conservation, whereas the effect of BMI was not significant.

**Table 3:** Multiple regression analysis predicting body shame.

	Men		Women	
Predictor	β	*t*	β	*t*
BMI (1 = healthy, 0 = overweight)	–0.27^∗^	–2.26	–0.12	–1.90
Self-enhancement	0.18	1.44	0.23^∗∗^	3.12
Conservation	0.11	0.72	0.17^∗^	2.22
Self-transcendence	–0.26	–1.66	–0.04	–0.44
Openness to change	0.09	0.61	–0.10	–1.24
^∗^*p* < 0.05, ^∗∗^*p* < 0.01	*R*^2^ adj. = 0.12		*R*^2^ adj. = 0.09	
	*F*(5,66) = 2.87, *p* < 0.05		*F*(5,244) = 5.72, *p* < 0.001	

**Table 4** reports findings about body surveillance. Self-enhancement was an important predictor for both men and women: participants who attributed priority to achieve personal success in society are those who were more engaged in body surveillance. Two other values acted as buffer against surveillance: in case of men giving importance to self-transcendence decreased the constant monitoring of appearance, while for women a similar role was played by openness to change.

**Table 4:** Multiple regression analysis predicting body surveillance.

	Men		Women	
Predictor	β	*t*	β	*t*
BMI (1 = healthy, 0 = overweight)	–0.03	–0.22	–0.00	–0.04
Self-enhancement	0.28^∗^	2.33	0.34^∗∗∗^	4.79
Conservation	0.22	1.50	–0.01	–0.18
Self-transcendence	–0.43^∗∗^	–2.72	0.03	0.29
Openness to change	0.25	1.83	–0.25^∗∗^	–3.27
^∗^*p* < 0.05, ^∗∗^*p* < 0.01, ^∗∗∗^*p* < 0.001	*R*^2^ adj. = 0.18		*R*^2^ adj. = 0.09	
	*F*(5,68) = 4.15, *p* < 0.01		*F*(5,244) = 6.03, *p* < 0.001	

## Discussion

Integrating objectification and value perspectives, the present study examined whether higher order values are related to self-objectification processes in both men and women.

About values, it is important to remember that the Schwartz model considers that values form a continuum (Cieciuch et al., 2014) and it is possible that a specific value is located on the border of two poles. Moreover, some cultural differences were described among European countries (op. cit., p. 11). Correlations among higher-order values are here all positive (see **Table 1**) because a value could “belong” to two different higher-order values (i.e., hedonism can be on the border of openness and self-enhancement values; or humility, located on the border of conservation and self-transcendence values – op. cit., p. 3).

The correlational analysis shows that the opposite poles have the lowest correlation index.

Taken together, findings support the conclusion that self-objectification may be fostered or discouraged by personal values, but some different results emerged between men and women and the considered dimension of self-objectification. Specifically, as hypothesized (Hypothesis 1), self-enhancement promotes self-objectification, increasing women’s body shame and men’s and women’s body surveillance. As seen, this value motivates people to follow personal interests and to achieve power according to socially prescribed standards (Schwartz, 1992; Cieciuch et al., 2014). We can argue that physical appearance could be considered as an indicator of personal worth and thus an essential basis for social realization. In this perspective, body surveillance becomes the necessary path to express through physical appearance the achievement of success and personal power. This phenomenon does not vary across male and female, in line with the arguments of Daniel et al. (2014) demonstrating the increasing tendency to objectify men as well in Western societies. However, experiencing shame as a consequence of self-enhancement might remain a women’s prerogative. In other words, body surveillance, in our society, is necessary to social success, but mostly for women a constant worry for having a body not compliant with cultural standards engenders the experience of shame.

Partially in line with our hypothesis (Hypothesis 2), conservation increased body shame in women. Conservative values probably include a more stereotypical conception of gender roles and characteristics. Women, and not men, holding traditional sexist attitudes may judge relevant the pursuit of physical attractiveness. Indeed, although beauty has been shown to play a key role in the consideration of both men and women (Langlois et al., 2000), traditionally women, more often than men, are socialized to the importance of “working on” their appearance and receive real reward that stem from fitting societal standards of beauty (Fredrickson and Roberts, 1997; Liss et al., 2011; Tartaglia and Rollero, 2015). Thus, women high in conservative values seem to be more prone to accept and internalize the “traditional duty” of adherence to cultural standards of appearance: when they feel not to be able, they experience shame.

The effects of self-transcendence and openness to change were not as protective as hypothesized (Hypotheses 3 and 4). The first higher order value was related to men’s body surveillance, whereas openness to change affected women’s body surveillance. In the Schwartz’s perspective (Schwartz, 1992; Cieciuch et al., 2014), self-transcendence refers to universalism and benevolence toward others, and it is opposed to self-enhancement. Findings concerning the male population are consistent with this bipolar conception: men focused on their own power achievement worry about their physical appearance as path to meet with their personal success, whereas men concerned with others’ welfare drive the attention to other issues and do not feel the necessity to constantly monitoring their appearance.

Results concerning women seem to reveal more intricate patterns. In their case, being high in self-transcendence values does not impact on self-objectification. However, women motivated in pursuing their own ideals, abilities, and interests, i.e., open to change, seem to challenge the objectifying cultural milieu: looking for a personal self-direction represents a protective factor against the internalization of objectification processes and thus decreases self-objectification. In line with results about self-enhancement, we can argue that sensitivity to social standards fosters self-objectification, whereas efforts to reach authenticity and freedom to cultivate personal interests prevents engagement in body surveillance. This pattern is near to the classical conceptualisation of Rogers (1961): according to him, authenticity can be conceived as the sense of empowerment and freedom to behave in a way which is an expression of personal principles, aims, and feelings, rather than the consequence of external expectations. In this sense, women who attribute priority to openness to change are less sensitive to external expectations, even those concerning their physical appearance.

Our data, in general, according to many studies (Strelan and Hargreaves, 2005) show that self-objectification is less strong for men; starting from this datum, we may argue that the negative consequences of sexual objectification are less strong for men as well (Saguy et al., 2010).

According to Loughnan et al. (2015), self-objectification affects both men and women, although the burden falls more heavily on women. But, to our knowledge, this was the first study which examined the role of higher order personal values on self-objectification, showing that personal values seem to act on self-objectification differently for male and female.

This study presents limitations and raises questions that are more than worthy of investigation by further research. The first limitation is that our research did not take into account other variables that can affect the relationship between values and self-objectification. Recent literature on values has claimed that contextual factors influence to what extent value motivations of individuals are expressed in their social attitudes: people high on conformity are more likely to regulate their behaviors to fit the specific normative context and downplay their personal values, whereas people lower in conformity are more likely to express their personal values in attitudes and behaviors (Boer and Fischer, 2013). Other ideological components may also be considered, such as endorsement of sexist attitudes, in line with the conception of self-objectification as a powerful cultural lens through which women view themselves, and through which to reiterate their own disadvantaged state (Calogero and Jost, 2011). Moreover, specific interaction analyses between sex and other relevant variables would extend knowledge about the role played by gender.

Another limitation is related to the involved population: the sample was restricted to white University students living in Italy and thus results may not be generalized to other groups of people. Future research should take into account specific characteristics of respondents, such as their age and educational level, as well as their cultural context. Moreover, we used self-report height and weight to compute BMI: although most studies in this field use these self-report measures, they represent a limitation as they rely on participants’ truthful.

Despite these limitations, we think this study offers some stimuli aimed to deepen the analysis of multi-factorial aspects that contribute to the development of self-objectification. In addition to implications related to research, we think that this study could offer some stimuli that can be used in training programs, especially for adolescents and youth people, aimed to develop values that should play an important role in contributing to, or protecting against, the development of self-objectification.

## Author Contributions

NDP and CR shared conception, design, and the final version of the work. NDP contribution was mainly in the theoretical part and in revising it critically. CR contributions was mainly in methodological question and data analysis. NDP and CR are jointly accountable for the content of the work, ensuring that all aspects related to accuracy or integrity of the study are investigated and resolved in an appropriate way. NDP and CR shared the internal consistency of the paper.

## Conflict of Interest Statement

The authors declare that the research was conducted in the absence of any commercial or financial relationships that could be construed as a potential conflict of interest.
